# Reply to Gumustepe, A.; Gumustepe, E. Comment on “Giordani et al. Cancer Association in Patients with Immune-Mediated Inflammatory Diseases: A Five-Year Nationwide Italian Cohort Study. *Cancers* 2026, *18*, 3449”

**DOI:** 10.3390/cancers18213518

**Published:** 2026-11-02

**Authors:** Barbara Giordani, Luigi Pirtoli, Guido Putignano, Antonio Giordano, Daniela Marotto, Giovanni Baglio

**Affiliations:** 1 Italian National Agency for Regional Healthcare Services (AGENAS), 00187 Rome, Italy; baglio@agenas.it; 2 Sbarro Institute, College of Science and Technology’s Biotechnology, Temple University, Philadelphia, PA 19122, USA; 3 Department of Medical Biotechnology, University of Siena, 53100 Siena, Italy; 4 bioERGOtech, 74123 Taranto, Italy; 5 Azienda Socio-Sanitaria Locale della Gallura, 07026 Olbia, Italy

We sincerely thank Gumustepe A. and Gumustepe E. for their careful reading of our study [1] and for their thoughtful comments [2], which give us the opportunity to clarify the scope and interpretation of our findings. We fully agree that the association between immune-mediated inflammatory diseases (IMIDs) and malignancy is complex and likely reflects the interaction of multiple biological, clinical and epidemiological mechanisms. At the same time, we consider it important to distinguish the objectives of our study from etiological questions that cannot be fully resolved using nationwide administrative hospital data.

Several of the raised issues—including residual confounding and the absence of detailed clinical information on disease activity, lifestyle factors and treatment exposure—correspond to limitations we had already reported, and the additional considerations regarding reverse causation and differential surveillance further refine the interpretation of the temporal pattern we observed. Our study was designed primarily to estimate the population-level burden and temporal pattern of cancer occurrence among patients with IMIDs in a nationwide real-world setting, rather than to establish a single underlying biological mechanism. Its distinctive contribution was not merely the increased occurrence of cancer (overall adjusted odds ratio [OR] 1.32, 95% CI 1.27–1.38), but the identification of a temporal gradient—a stronger association during early follow-up (OR 1.83, 95% CI 1.61–2.08 in year one) followed by progressive attenuation to a persistent excess thereafter (OR 1.20, 95% CI 1.15–1.30 from year five)—which represents an important hypothesis-generating observation at the national level. We address the specific points raised below.

## 1. Reverse Causation and Paraneoplastic Autoimmunity

We agree that the association between cancer and autoimmunity is bidirectional, and that reverse causation and paraneoplastic autoimmunity may account for some cancers diagnosed shortly after cohort entry. This is particularly relevant to specific rheumatic conditions, such as idiopathic inflammatory myopathies, in which malignancy frequently clusters around disease onset and cancer-associated myositis is well established [3,4]. However, our cohort included a broad spectrum of IMIDs, most of which do not share a paraneoplastic association of comparable magnitude. Occult malignancy-associated immune activation may therefore explain a proportion of the cancers diagnosed during early follow-up, but this mechanism cannot be generalized to the entire IMID population.

More importantly, reverse causation and inflammation-related mechanisms need not be regarded as competing explanations. The association between autoimmunity and malignancy is increasingly understood as bidirectional and multifactorial, potentially involving chronic inflammatory stimulation, immune dysregulation, shared genetic and environmental susceptibility, treatment-related effects and, in selected patients, tumor-induced immune phenomena. The early cancer peak most plausibly reflects a combination of these mechanisms rather than a single causal pathway.

## 2. Surveillance and Detection Bias

We likewise agree that enhanced diagnostic surveillance is a plausible contributor to the excess detected during the first year of follow-up. Patients with IMIDs undergo laboratory investigations, imaging and specialist assessments more frequently than individuals without chronic inflammatory disease, which increases the probability of detecting otherwise asymptomatic or subclinical malignancies; this is especially relevant given that our comparison group comprised patients hospitalized for injury and poisoning, who may not have undergone comparable longitudinal surveillance. Crucially, however, the increased cancer occurrence was not confined to the initial period but persisted, although attenuated, through the fifth year (OR 1.20, 95% CI 1.15–1.30). Detection bias or prevalent occult malignancy alone would be expected to produce an early excess that resolves once the initial diagnostic backlog is exhausted, rather than the sustained association we observed. Intensified surveillance, reverse causation and underlying disease-related mechanisms should therefore be regarded as complementary rather than mutually exclusive, and methodological uncertainty regarding the mechanism should not be conflated with uncertainty regarding the epidemiological pattern itself, which is robust.

## 3. Cohort Entry and Disease Duration

Gumustepe A. and Gumustepe E. correctly note that cohort entry corresponded to the index hospitalization in 2018 and did not necessarily coincide with the biological onset or first diagnosis of the IMID. In our paper, we explicitly acknowledged the limitation arising from the lack of a precise disease onset timing in interpreting the results. Nevertheless, the biological onset of chronic immune-mediated diseases is difficult to establish even in clinically enriched datasets and cannot be reliably reconstructed from administrative hospital records. The objective of our study was not to reconstruct the natural history of carcinogenesis from the onset of each individual IMID, but to compare cancer occurrence in a large nationwide group of patients with documented IMIDs against the unexposed group. We agree that studies specifically restricted to well-characterized incident IMIDs represent the appropriate next step and would provide valuable complementary evidence.

## 4. Interpretation of the Temporal Pattern and the Role of Inflammation

In our paper we stated that the progressive decline in the association over time generates the hypothesis that inflammation, rather than medication, may be a primary driver of the observed association. This wording was deliberately hypothesis-generating rather than causal, consistent with the primary aim of estimating the association between IMIDs and first cancer occurrence at the population level, both overall and by the cancer site. We agree that the temporal pattern cannot, by itself, establish a causal role for systemic inflammation.

The temporal gradient is compatible with several overlapping mechanisms, including chronic inflammation and immune dysregulation, but also reverse causation, occult malignancy-associated autoimmunity, enhanced diagnostic surveillance, heterogeneous disease duration, treatment exposure and shared environmental or lifestyle factors. We therefore accept that the early peak should not be read as direct evidence of inflammation-driven carcinogenesis, but rather as an epidemiological observation that generated a biologically plausible hypothesis requiring confirmation in studies specifically designed to test causality. At the same time, the persistence of excess beyond the initial period argues against attributing the entire cancer burden to early detection, differential surveillance or pre-existing occult malignancy. The most defensible interpretation is thus not the substitution of one single causal explanation for another, but the recognition that the association is likely multifactorial and may differ substantially according to the specific IMID and cancer type—implying that cancer risk in IMIDs is dynamic and may reflect different predominant mechanisms at different stages of follow-up.

## 5. Treatment Exposure

We agree that, in the absence of detailed longitudinal information on disease activity, inflammatory markers and treatment exposure, the declining association over time cannot be interpreted as evidence of improved inflammatory control or of a protective effect of anti-inflammatory therapy, and we do not present it as such. Equally, our findings should not be taken to demonstrate that treatment has no role in malignancy risk: the oncological effects of immunomodulatory therapies are heterogeneous and depend on the specific agent, the underlying disease, patient characteristics, the duration of exposure and the cancer type. Neither cumulative treatment exposure nor inflammation can be isolated from the present data; rather, our results are consistent with a multifactorial framework in which disease-related inflammation and immune dysregulation coexist with treatment exposure, shared susceptibility, reverse causation and differential surveillance.

## 6. Smoking, Environmental Exposures, and Site-Specific Associations

We agree that the absence of individual-level smoking data is an important limitation, particularly for the interpretation of lung cancer risk. Smoking is a major determinant of lung cancer and is associated with selected inflammatory diseases, notably seropositive rheumatoid arthritis, making it a potentially important source of residual confounding; ever-smoking patients with rheumatoid arthritis have been reported to carry a markedly higher lung cancer risk than never-smoking controls [5,6]. Because adjustment for chronic respiratory diagnoses cannot substitute for direct information on smoking status, cumulative pack-years or cessation history, the association with lung cancer should be interpreted with appropriate caution. This consideration reinforces, rather than undermines, our argument against a uniform etiological interpretation across malignancies. Hematological malignancies may plausibly reflect chronic B-cell activation and immune dysregulation [7], whereas lung and bladder cancers are substantially influenced by environmental and lifestyle exposures [8]. This heterogeneity is consistent with the broader epidemiology of IMIDs and with our own site- and subtype-specific estimates, in which risk varied by both the underlying disease and the cancer type.

## 7. Sensitivity Analyses and Future Research

We welcome the suggestion to carry out sensitivity analysis excluding cancers diagnosed during the first 12–24 months of follow-up, even though the analyses for different time intervals reported in our article already partly address this request. Similarly, restricting the analysis to incident, well-defined autoimmune diseases would provide valuable complementary information but requires reliable identification of true disease onset, which is not consistently attainable from hospital data.

We agree that future prospective cohorts of well-characterized incident IMIDs—incorporating data on disease onset, activity, biomarkers, treatment, lifestyle and healthcare utilization—would allow the relative contributions of inflammation, immune dysregulation, treatment, reverse causation and surveillance bias to be disentangled. Nonetheless, clinically enriched cohorts and nationwide studies based on administrative data should be regarded as complementary, rather than competing approaches. In fact, the hospital information system provides exceptional population coverage, statistical power and remains particularly valuable for studying disease-specific cancer associations that would be difficult to detect in smaller clinical cohorts.

## 8. Conclusions

We thank Gumustepe A. and Gumustepe E. for highlighting important considerations. Their comments refine, rather than deny, our findings.

We believe that the inherent weakness of health information system-based research in confirming causal hypotheses does not diminish the evidence provided by this Italian nationwide cohort study. Indeed, the comments reinforce its value by outlining the range of biological and epidemiological mechanisms that should be further investigated.

## Data Availability

The data are available from AGENAS with its permission and upon reasonable request, by contacting direzione.ricerca@agenas.it.
